# Real World Case Series: Integrated Skincare With Advanced RGN‐6 Serum

**DOI:** 10.1111/jocd.70303

**Published:** 2025-07-25

**Authors:** Andrew Alexis, Renée A. Beach, Patricia Brieva, Sophie Guénin, Omar A. Ibrahimi, Michelle Rodrigues, Heather Woolery‐Lloyd, Valerie Callender

**Affiliations:** ^1^ Weill Cornell Medicine New York New York USA; ^2^ DermAtelier on Avenue Faculty of Medicine University of Toronto Toronto Ontario Canada; ^3^ SkinCeuticals New York New York USA; ^4^ Mount Sinai Dermatology New York New York USA; ^5^ Connecticut Skin Institute Milford Connecticut USA; ^6^ Chroma Dermatology, Clinical Department of Dermatology Royal Children's Hospital Parkville Victoria Australia; ^7^ Skin of Color Division Dr Phillip Frost Department of Dermatology and Cutaneous Surgery University of Miami Miller School of Medicine Miami Florida USA; ^8^ Howard University College of Medicine, Callender Dermatology & Cosmetic Center Glenn Dale Maryland USA

**Keywords:** aging face, cosmeceuticals, mature skin

## Abstract

**Background:**

Skin aging is a multifactorial process with intrinsic and extrinsic factors that lead to visible signs of aging, including loss of skin elasticity, volume, dyspigmentation, wrinkles, fine lines, and dry and uneven skin. The advanced RGN‐6 serum is a patent‐pending, multi‐ingredient serum that has been developed to address 6 dimensions of skin regeneration and combat visible signs of aging. The six dimensions of skin regeneration include: (1) barrier re‐epithelialization, (2) redness and inflammation, (3) cellular energy stimulation, (4) elastin and collagen stimulation, (5) antioxidants, and (6) postinflammatory hyperpigmentation (PIH). To address these factors, the RGN‐6 serum contains six active ingredients: 1% eperuline, 0.2% ectoin, 10% glycorepair, 0.2% bioceramide 603, 3% acetyl tetrapeptide‐9, and 2% niacinamide. This multi‐ingredient serum works to simultaneously target the most common signs of aging.

**Aim:**

To provide detailed evidence for multiple integrated skincare regimens using the advanced RGN‐6 serum in facial rejuvenation.

**Methods:**

In this real‐world case series, 6 expert dermatologists with extensive experience in cosmetic and anti‐aging medicine shared and discussed patient cases of advanced RGN‐6 serum used in combination with energy‐based facial rejuvenation procedures in a subset of women with skin of color with Fitzpatrick skin types ranging from type 3 to type 6.

**Results:**

After the panel discussion, six patient cases were selected to best demonstrate the use of RGN‐6 serum postprocedure.

**Conclusion:**

The panel experts agreed that twice daily application of the RGN‐6 serum led to improved signs of redness, fine lines, and skin tone and evenness in all postprocedure patients.

## 1 Introduction

Skin aging is a complex process that has significant psychosocial impacts on an individual. Extensive research has been conducted to develop a multitude of products that have led to a growing global anti‐aging market reflective of the increasing demand from an aging population [1]. The aging process consists of both extrinsic and intrinsic factors that lead to biological and clinical signs of aging [2].

Extrinsic factors consist largely of sun exposure (UV light), air pollution, tobacco smoke, nutrition, stress, sleep, and various cosmetic products [3]. UV radiation, or photoaging, has been well established as an important driver of skin aging [3]. Photoaging primarily occurs in the dermal layer of the skin where age‐related remodeling leads to wrinkles and changes to skin texture and firmness. Collagen is an important part of skin architecture and serves as the scaffold for extracellular matrix (ECM) components that draw in hydration and maintain skin smoothness and firmness [1, 2]. Loss of collagen occurs over time in part due to UV‐activated matrix metalloproteinases (MMP) that lead to collagen degradation and reorganization [1, 2]. In addition, DNA damage triggered by physiological aging, UV radiation, pollution, and tobacco smoke also contributes to appearance of skin roughness, fine lines, and wrinkles [1, 2]. UVA light is recognized as a key driver of DNA damage via generation of free radicals and reactive oxygen species (ROS), which stimulate inflammation in the skin and lead to skin thickening, dyspigmentation, redness and telangiectasias [1]. ROS leads to release of proinflammatory cytokines interleukin‐1 (IL‐1) and tumor necrosis factor‐α (TNF‐ α) that directly cause DNA breakage, depletion of antioxidants and generation of other inflammatory mediators such as histamine and leukotrienes, leading to redness and further inflammation [4]. Lifestyle and skincare practices such as eating a well‐balanced diet abundant in antioxidants and using protective sunscreens can help protect and counteract the impact of extrinsic aging factors [1].

Intrinsic factors of aging refer to the natural, genetically programmed process of chronological aging [1]. Flattening of the dermal–epidermal junction (DEJ) is a characteristic sign of skin aging that occurs due to loss of collagen and elastin fibers over time [1, 5]. DEJ flattening leads to a reduction in skin barrier integrity and structure [1]. Hormonal changes such as a decline of estrogen in women and testosterone in men lead to decreased collagen production, skin hydration, and subsequent wrinkle formation [5]. Metabolic processes also slow with age, which leads to a buildup of cellular waste that can produce advanced glycation end products (AGEs), which are glycated proteins or lipids that can crosslink collagen fibers, making them stiff, less elastic, and contributing to wrinkle and fine line appearance [5]. Further, genetic predisposition can also significantly impact an individual's aging process, as specific genes involved in cellular repair, oxidative stress responses, and collagen and elastin production may vary widely between individuals [5].

**FIGURE 1:**
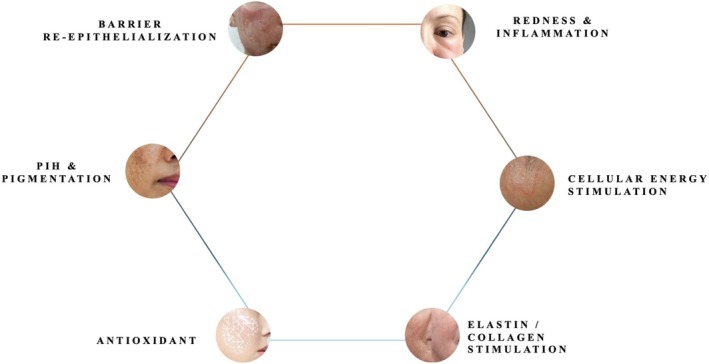
Six dimensions of skin rejuvenation and anti‐aging. The RGN‐6 serum simultaneously targets the 6 features of skin rejuvenation: (1) barrier re‐epithelization, (2) redness and inflammation, (3) cellular energy stimulation, (4) elastin and collagen stimulation, (5) antioxidant, and (6) postinflammatory hyperpigmentation (PIH).

Considering extrinsic and intrinsic factors of aging, the most common reported signs of aging include loss of skin firmness and elasticity, dyspigmentation (including hyperpigmentation, redness, and telangiectasias), wrinkles, and fine lines. These changes are largely a result of decreased collagen and elastin in the skin. The Advanced REGEN (RGN)‐6 corrective serum is an aesthetic treatment that rebuilds the skin to reverse visible signs of aging. The RGN‐6 serum contains six active ingredients that trigger the six dimensions of skin regeneration: (1) barrier re‐epithelization, (2) inflammation, (3) cellular energy stimulation, (4) elastin and collagen stimulation, (5) antioxidant, and (6) postinflammatory hyperpigmentation (PIH) (Figure 1). The serum targets the same aspects of skin aging as many energy‐based devices and chemical peels in the common goal of reversing signs of skin aging. This suggests that the topical could be used in conjunction with facial regeneration procedures such as nonablative fractional laser, ablative CO2 laser, and microneedling radiofrequency (RF) to produce enhanced patient results. The serum contains six active ingredients: 0.1% eperuline, 0.2% ectoin, 10% glycorepair, 0.2% bioceramide 603, 3% acetyl tetrapeptide‐9, and 2% niacinamide. Eperuline and ectoin are antioxidant and anti‐inflammatory ingredients that help combat photoaging [6]. Glycorepair, bioceramide 603, and acetyl tetrapeptide‐9 help to support epidermal recovery and stimulate collagen and elastin production in the dermis [7]. Lastly, niacinamide is present to target PIH and dyspigmentation [8]. The active ingredients and their actions can be found in Table 1.

**TABLE 1:** Advanced RGN‐6 serum active ingredients.

Ingredient	Derivative	Action
Eperuline	Derived from Eperua falcata bark extracted from trees within the Guyanese rainforest, rich in tannins and flavonoids, and has been used medicinally by local Amerindians to alleviate discomfort in wound healing.	Antioxidant Stimulates cellular energy for the removal of damaged cells
Ectoin	Derived from an extremolyte that helps organisms to live under extreme environmental stress conditions (UV‐stress, heat, dryness), maintain cell stability, and provide protein and cell protection.	Antioxidant protection (protects from UVA stress) Anti‐inflammatory (reduces in vitro levels of proinflammatory TNF‐a, IL‐1a, and IL‐8)
Glycorepair	Derived from carob seed extract, which helps to repair and reactivate the biological pathways involved in skin re‐epithelialization	Accelerates migration capacity of keratinocyte and inhibits elastase; thereby increasing epidermal and dermal regeneration
DVS603	A nature‐inspired biomimetic ceramide (ceramide 2 analogue) co‐created by L'Oreal to reduce melanogenesis and provide barrier function reinforcement	Prevents pigmentation via reduced gene expression of melanogenesis enzymes Stimulates barrier repair
Acetyl Tetrapeptide‐9 (Dermican)	A pure tetrapeptide to restructure the extracellular matrix by stimulating the synthesis of lumican and collagen—improving skin thickness and firmness	ECM protein stimulation Increase in skin firmness and thickness
Niacinamide	Vitamin B3, a water‐soluble vitamin	Strengthens skin moisture barrier reduces dyschromia and postinflammatory hyperpigmentation

Here, we present six real‐world patient cases to highlight experience with an integrated skincare regimen using the advanced RGN‐6 serum in conjunction with facial rejuvenation procedures.

## 2 Methods

### 2.1 Aim of the Project

This real‐world case series was composed to highlight integrated skincare regimens using the advanced RGN‐6 serum in conjunction with facial rejuvenation procedures such as lasers and microneedling. These cases aim to illustrate how expert dermatologists employ the RGN‐6 serum to optimize patient outcomes after regenerative procedures in the clinic. Expert panelists' reasoning and rationale are outlined in the presented cases to guide dermatologists and patients seeking to construct integrated skincare regimens for maximum skin rejuvenation. These cases aim to illustrate the use of the serum; however, they do not represent results from a placebo‐controlled clinical trial.

### 2.2 Steps in the Process

The real‐world cases were compiled and selected in the following steps: 1) project definition and expert panel selection, 2) data collection and preparation of patient cases, 3) patient case discussion and selection for publication, 4) literature review to support selected cases, 5) drafting, review, and finalization of the manuscript.

### 2.3 Role of the Panel

The selected expert panel consisted of 6 experts, board‐certified dermatologists with extensive experience in cosmetic and anti‐aging medicine. Experts were selected from various clinical care settings, including urban, suburban, academic, and nonacademic settings. Panelists represented three countries, including Canada, Australia, and the United States, and were selected based on their experience treating diverse patient populations. They were selected to provide a greater understanding of integrated skincare practices in a wide variety of patients and skin types across different countries and care settings. Panelists met on September 14th, 2024, in New York, New York, during the annual Skin of Color Update conference to discuss patient cases using the advanced RGN‐6 serum in their cosmetic practices.

The panel used the following template to gather insight through a case‐based approach:

1. Cosmetic evaluation and alignment of treatment goals
2. Facial rejuvenation treatment plan with integrated advanced RGN‐6 serum use
3. Physician clinical assessment
4. Patient self‐assessment
5. Special considerations and key takeaways

Each panelist shared 2 cases for a total of 10 cases presented during the meeting. After discussion with the meeting chairs, Drs. Andrew Alexis and Valerie Callender, 6 real‐world cases were selected to be presented in this manuscript. These cases were chosen as they best highlighted the serum's use in postprocedure settings to optimize patient results.

### 2.4 Advanced RGN‐6 Serum

Patients were provided with advanced RGN‐6 Serum (SkinCeuticals, USA) to be used twice daily on their full face for 4 weeks following their procedure along with SkinCeuticals support products: Simply Clean cleanser and Sheer Physical UV Defense SPF 50. On the day of their baseline visit and procedure, patient skin was cleansed with Simply Clean cleanser followed by the use of the selected energy‐based device or chemical peel. After the selected procedure, the patient was instructed to apply the advanced RGN‐6 serum twice daily starting that evening. All patients were instructed to use SkinCeuticals Sheer Physical UV Defense SPF 50 daily to protect skin from UV damage.

#### 2.4.1 Active Ingredients

Eperua Falcata bark extract (Eperuline), *Ceratonia Siliqua* (Carob) seed extract (Glyco‐repair), 2‐oleamido‐1,3‐octadecanediol (DVS603), Acetyl tetrapeptide‐9, Niacinamide, 1,4,5,6‐tetrahydro‐2‐methyl‐4‐pyrimidinecarboxylic acid (Ectoin).

#### 2.4.2 Full Ingredient List for advanced RGN‐6 serum

Water, glycerin, ethyl oleate, niacinamide, squalane, C12–16 alcohols, glyceryl stearate citrate, butyrospermum parkii butter/shea butter, maltodextrin, ammonium polyacrylodimethyl taurate, phenoxyethanol, dimethicone, palmitic acid, hydrogenated lecithin, cetyl alcohol, *Ceratonia siliqua* seed extract/carob seed extract, caprylyl glycol, 2‐oleamidio‐1,3‐octadecanediol, ectoin, tocopherol, eperua falcata bark extract, xanthan gum, ethylhexylglycerin, phytic acid, adenosine, sodium hydroxide, sodium benzoate, acetyl tetrapeptide‐9, and citric acid.

### 2.5 Data Gathering and Outcome Measures

Suggested information to present included patient demographics, skin type, and facial rejuvenation procedure selected. Patients were followed up for 4 weeks with visits at baseline, day 1, week 1, week 3, and week 4. At each visit, physician assessment scores were recorded for the following categories: fine lines, wrinkles, firmness, elasticity, laxity, radiance, and smoothness. The scores were determined using the 10‐point modified Griffiths scale for photodamage (0 = none, 1–3 = mild, 4–6 = moderate, 7–9 = severe), using facial photodamage and repair as a surrogate for facial rejuvenation in specified categories [9]. Physician assessment also included a 5‐point global aesthetic improvement scale (GAIS) (1 = very much improved, 2 = much improved, 3 = improved,4 = no change, 5 = worse) evaluated at day 1, week 1, week 3, and week 4. Physician evaluation of skin dryness and erythema on a 5‐point scale (0 = none, 1 = minimal, 2 = mild, 3 = moderate, 4 = severe) was also included at each visit. Lastly, discomfort, tolerability, and adverse events were noted at each visit. Qualitative responses were also collected from patients at each follow‐up visit in a self‐assessment questionnaire. Patients were asked to rate 15 statements on a 5‐point Likert scale (5 = strongly agree, 4 = agree, 3 = neutral, 2 = disagree, and 1 = strongly disagree). Examples of the physician assessment and subject assessment templates can be found in Tables 2 and 3, respectively. Photography was taken, if possible, at Visit 1 (Baseline/Preprocedure, Postprocedure+ Product Application), Visit 2, Visit 3, Visit 4, and Visit 5.

**TABLE 2:** Physician assessment template.

	Fine Lines	Wrinkles	Firmness	Elasticity	Laxity	Radiance	Smoothness	Overall appearance	Discomfort/Tolerability	Dryness	Erythema	Corneometer Instrumental (Optional)
Panelist ID:_____________ Age:______ FITZ:____ Gender (F/M):___ Skin Type:______ Procedure:	Modified Griffith's Scale 0–9 (10‐point scale) 0 = None (best possible condition) 1–3 = mild 4–6 = moderate 7–9 = severe (worst possible condition)							GAIS (5 point scale: 1–5) 1 = very much 2 = much improved 3 = improved 4 = no change 5 = worse	Yes or No	5 point Scale: (0–4) 0 = none 1 = minimal 2 = mild 3 = moderate 4 = severe		Make/Model
	Score (0–9)	Score (0–9)	Score (0–9)	Score (0–9)	Score (0–9)	Score (0–9)	Score (0–9)	Score (1–5)	Score (yes or no)	Score (0–4)		Instrumental
Visit 1: Baseline/Preprocedure												
Visit 2: Postprocedure+ Product Application Day 1												
Visit 3: Week 1												
Visit 4: Week 3												
Visit 5: Week 4												

**TABLE 3:** Subject assessment template.

	Questions	Visit 2	Visit 3	Visit 4
1	Product is suitable to use postprocedure.			
2	Product has a pleasant texture.			
3	Skin feels smoother.			
4	Skin feels firmer.			
5	Skin feels moisturized/hydrated.			
6	Skin feels protected.			
7	Skin appears tighter.			
8	Skin appears renewed.			
9	Skin appears younger.			
10	Skin appears to have a healthy glow.			
11	Visible fine lines are reduced.			
12	Visible wrinkles are reduced.			
13	Overall, appearance of skin is improved.			
14	Overall, how satisfied are you with the product?			
15	I feel more ready to do another procedure.			

*Note:* 5‐point scale: 5—Strongly Agree, 4—Agree, 3—Neutral, 2—Disagree, 1—Strongly Disagree.

## 3 Results

Six patient cases were selected to demonstrate use of the advanced RGN‐6 serum in conjunction with facial rejuvenation procedures as part of integrated skincare regimens. The cases represented use of the serum after procedures in women (Fitzpatrick Skin Types (FST) 3–6). Selected cases are summarized in Table 4.

**TABLE 4:** Summary of selected real‐world patient cases.

Case #	Demographics	Concerns	Procedure	Key Features
1	77F FST 5	Skin laxity, forehead wrinkles age‐related dyschromia melasma, hyperpigmentation	Salicylic acid Peel	Improvement in wrinkles, UV spots, and porphyrins
2	71F FST 5	Combination skin loss of firmness	Sofwave ultrasound	Improvement in skin radiance and evenness
3	28F FST 5	Concerns of skin discoloration, PIH, acne scarring	RF microneedling	Safety in acne prone skin
4	55F FST 3	uneven, dull, photoaged skin, dark spots, dyschromia	Picoway laser spot treatment	Significant reduction in erythema
5	42F FST 3	Skin discoloration, multiple dark spots/sunspots	Picoway laser spot treatment	No change in skin condition with RGN‐6 serum
6	49F FST 6	Clinical hyperpigmentation	Aerolase LightPod Neo	Improvement in hyperpigmentation

### 3.1 Case 1. Salicylic Acid Peel and Advanced RGN‐6 Serum to Target Hyperpigmentation

**FIGURE 2:**
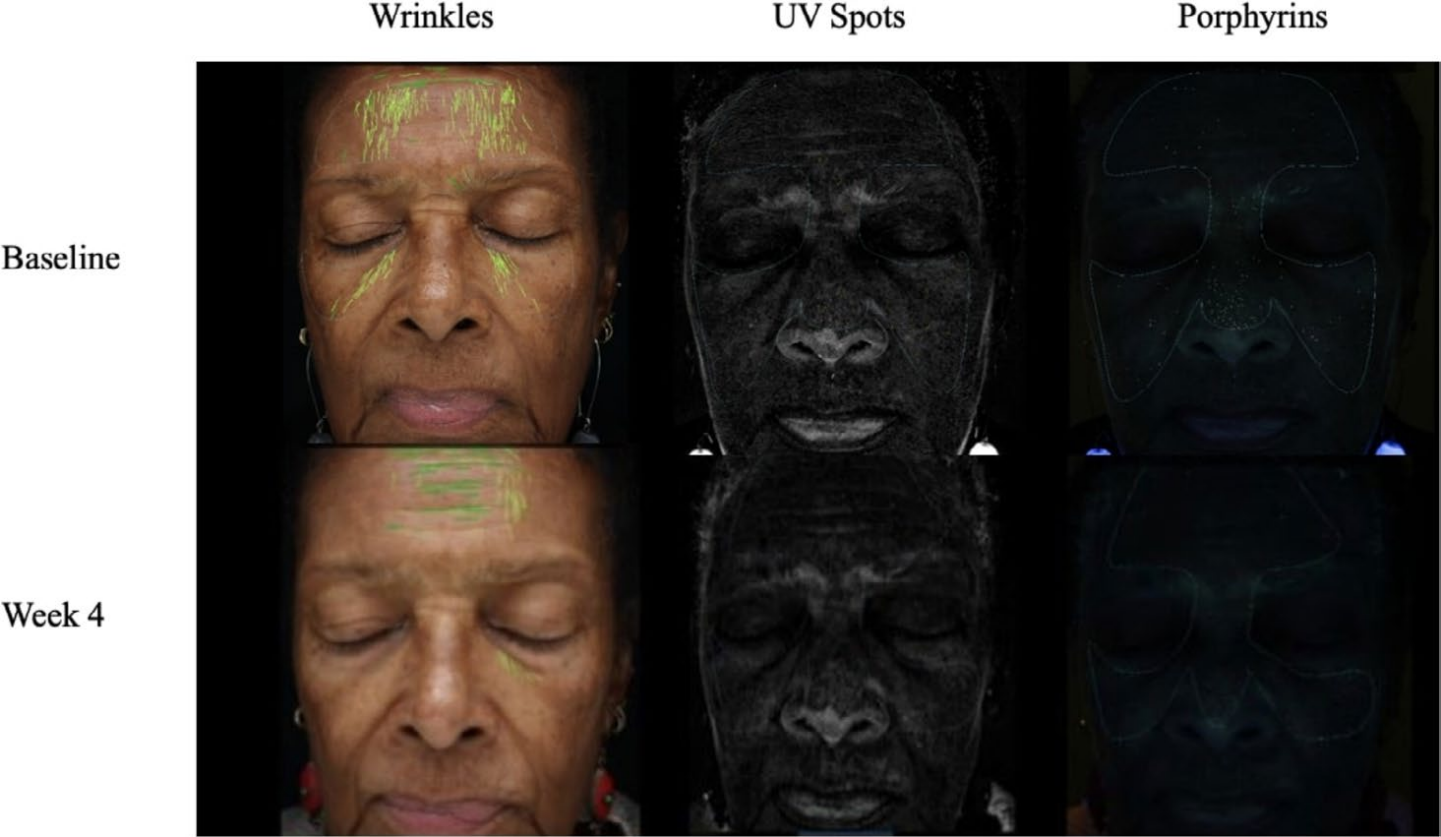
Case 1. A 77‐year‐old woman (Fitzpatrick Skin Type 5). Use of salicylic acid peel in conjunction with advanced RGN‐6 serum.

A 77‐year‐old woman, FST 5, presented with skin laxity, forehead wrinkles and concerns of age‐related dyschromia, melasma and hyperpigmentation. At baseline, the physician assigned a score of 6 for fine lines, 8 for skin tone/evenness, 8 for skin clarity, 5 for firmness, 5 for elasticity, 6 for radiance, and 4 for overall smoothness. Subsequently, a salicylic acid peel was chosen to help best target this patient's concerns of hyperpigmentation and uneven skin tone. The patient was then instructed to apply the RGN‐6 serum twice daily to the face to optimize postpeel results. Used in conjunction with the peel, the RGN‐6 serum was hypothesized to provide antioxidant and anti‐inflammatory effects postpeel that would allow the patient to achieve optimal results with a reduction in recovery time. After 1 week of serum use, the patient returned for a follow up visit and noticed improvement in fine lines, skin tone/evenness, skin clarity, and smoothness. The patient continued to see improvement and by week 4 had a score of 3 for fine lines, 6 for skin tone/evenness, 6 for skin clarity, 5 for skin firmness, 5 for skin elasticity, 5 for radiance, and 3 for skin smoothness. A final GAIS score of 3 was given at week 4 (Figure 2). Using facial skin analysis techniques, appearance and severity of patient wrinkles, pores, UV spots, brown spots, red areas, and porphyrins were analyzed at baseline and week 4. By week 4, analyses revealed that there was a 16% improvement in wrinkle percentile ranking, 26% improvement in UV spots percentile ranking, and 42% improvement in porphyrin percentile ranking without any measurable change in pores, brown spots, and red areas (Figure 2). The RGN‐6 serum was well tolerated, and the patient did not report any discomfort and felt that her skin was smoother and more hydrated.

### 3.2 Case 2. Combination of Sofwave Ultrasound Use With Advanced RGN‐6 Serum in Facial Rejuvenation

**FIGURE 3:**
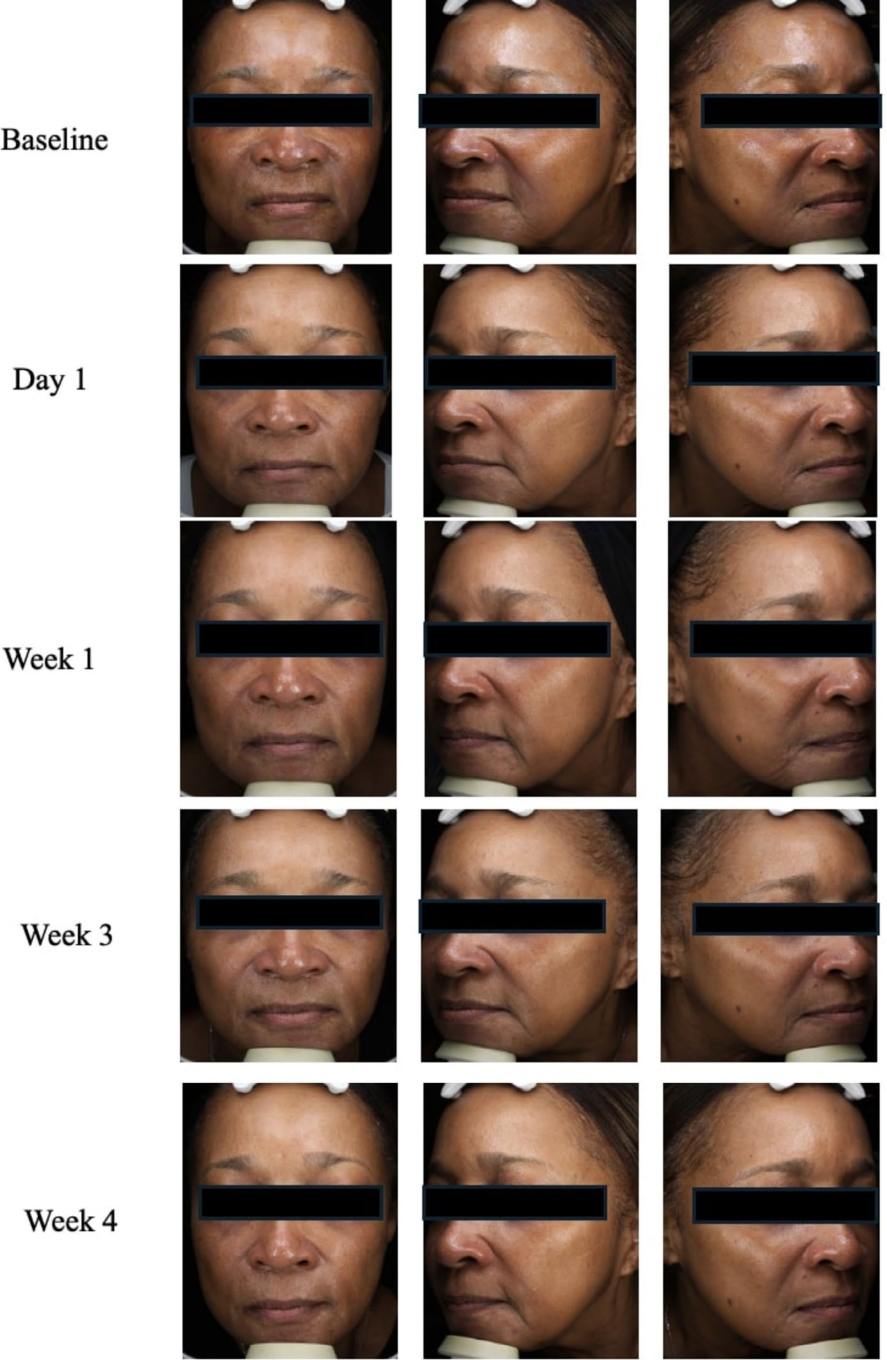
Case 2. A 71‐year‐old woman (Fitzpatrick Skin Type 5). Use of Sofwave in conjunction with advanced RGN‐6 serum.

A 71‐year‐old woman, FST 5, presented with combination skin and loss of skin firmness and youth. The Advanced RGN‐6 serum was selected in conjunction with the Sofwave ultrasound treatment to provide a multitargeted approach to skin rejuvenation. At baseline, physician assessment skin scores were the following: 4 for fine lines, 2 for skin tone/evenness, 2 for skin clarity, 5 for skin firmness, 5 for skin elasticity, 3 for radiance, and 5 for skin smoothness. The patient only saw significant results at week 4 and had a GAIS score of 2, much improved (Figure 3). Physician skin assessment scores at week 4 for were the following: 3 for fine lines, 1 for skin tone/evenness, 1 for skin clarity, 3 for skin firmness, 3 for skin elasticity, 1 for radiance, and 2 for smoothness. The RGN‐6 serum was well tolerated without any reported adverse effects. The patient was also highly satisfied with the adjunctive treatment and felt that her skin had a healthier glow and appeared younger than before.

### 3.3 Case 3. RF Microneedling and Advanced RGN‐6 Serum in Acne Prone Skin

**FIGURE 4:**
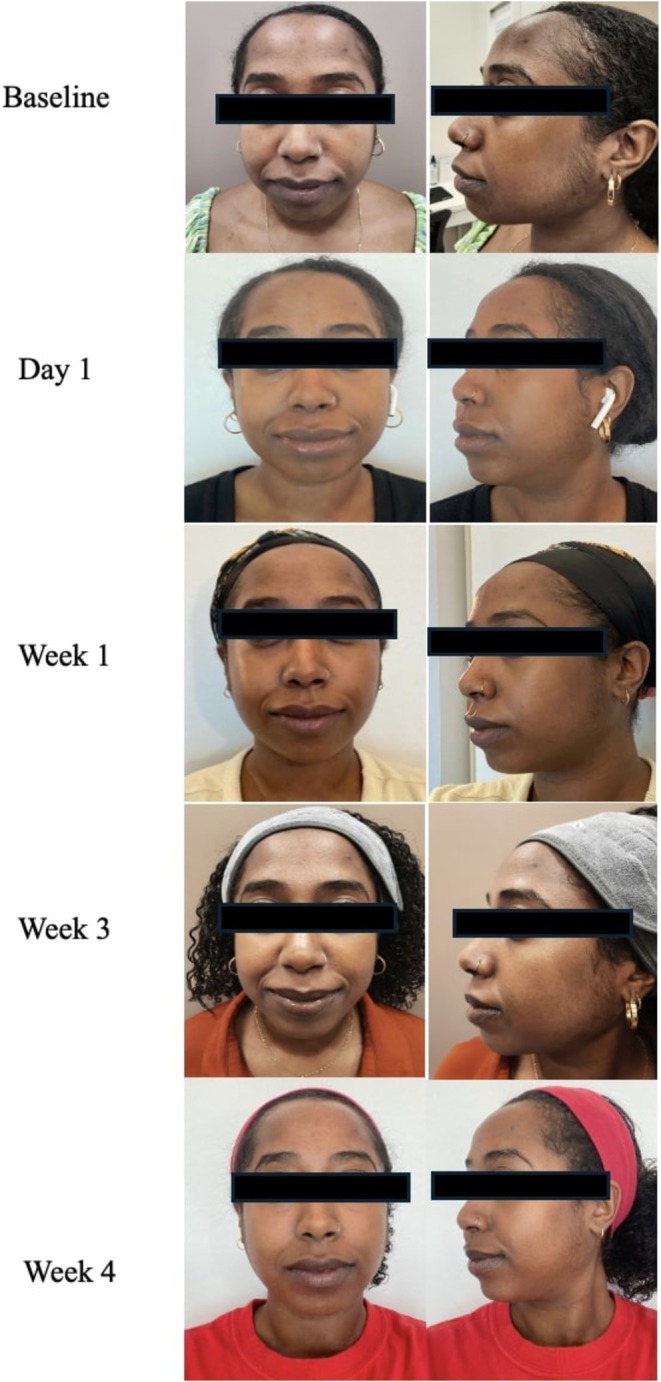
Case 3. A 28‐year‐old woman (Fitzpatrick Skin Type 5). Use of RF microneedling in conjunction with advanced RGN‐6 serum.

A 28‐year‐old woman, FST 5, presented with concerns of skin discoloration, PIH, and acne scarring. She had notably acne‐prone skin, which was an important factor in considering adjunctive skincare for an optimal outcome. The patient underwent RF microneedling at her baseline visit to help with skin lifting, texture, and dyspigmentation. The advanced RGN‐6 serum was discussed with the patient for twice daily use to complement this procedure. The patient was enthusiastic about trying an adjunctive treatment to provide added benefits to her skin. At baseline, her physician assessment scores were 3 for fine lines, 5 for skin tone/evenness, 4 for skin clarity, 3 for firmness, 3 for elasticity, 4 for radiance, and 3 for smoothness. The patient saw rapid improvement in her skin and by her day 1 visit, she had a 2‐point reduction in fine lines, skin tone/evenness, elasticity, and radiance. By week 4, the patient had a score of 1 for fine lines, skin tone/evenness, firmness, elasticity, radiance, and smoothness with a score of 0 for skin clarity. The patient did not report any discomfort with the application of the serum at any point during the treatment period and strongly agreed that the serum provided an added benefit to her treatment plan. Final GAIS was a 3 indicating improvement since baseline (Figure 4). She felt that her overall skin had improved over the 4 weeks and felt that her skin tone and evenness had been restored.

### 3.4 Case 4. Picoway Laser Treatment With Advanced RGN‐6 Serum for Dark Spots and Toning

**FIGURE 5:**
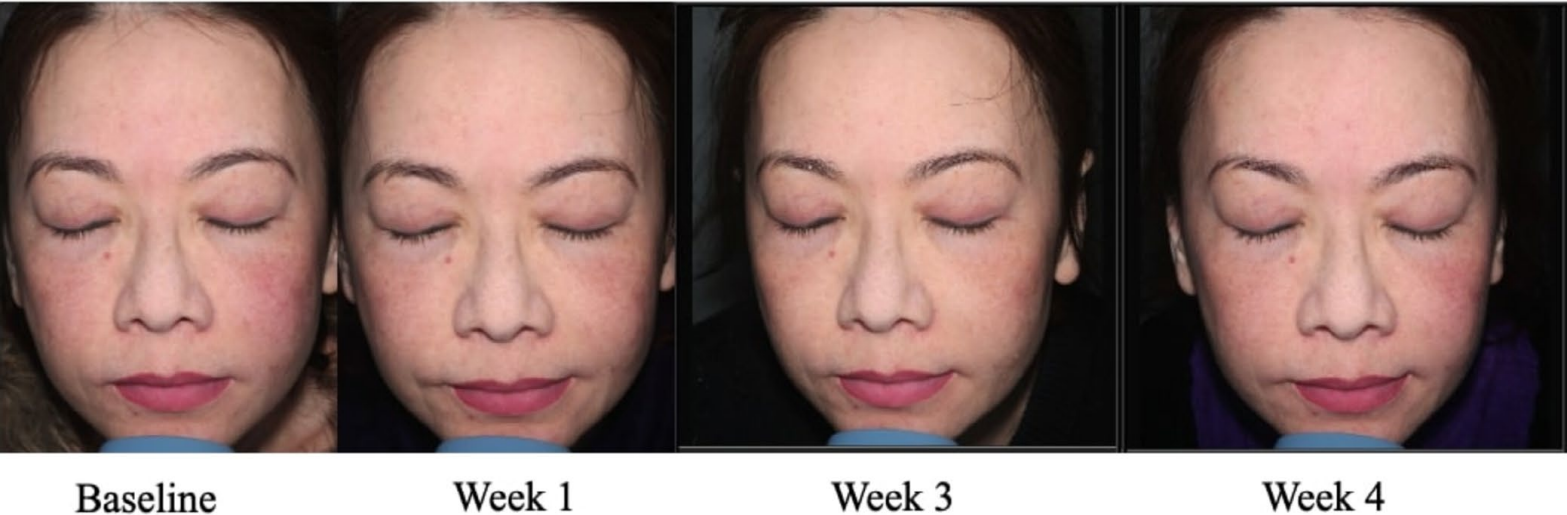
Case 4. A 55‐year‐old woman (Fitzpatrick Skin Type 3). Use of Picoway laser treatment in conjunction with advanced RGN‐6 serum.

A 55‐year‐old female, FST 3, presented with uneven, dull‐looking, photoaged skin. She was particularly concerned about multiple dark spots and dyschromia on her face. The expert dermatologist decided to use the Picoway laser to spot treat the multiple dark spots on her face. Following Picoway laser spot treatment, the patient was instructed to use the advanced RGN‐6 serum to continue topical rejuvenation treatment. At baseline, the patient's most severe scores were an 8 for skin tone and evenness and 6 for skin clarity. By day 1 post treatment, the patient already had significantly improved with a 5‐ and 3‐point improvement in skin tone/evenness and clarity, respectively. By week 4, the patient had a score of 2 for skin tone/evenness and skin clarity with scores of 1 for fine lines, firmness, elasticity, and radiance. The patient did not report any discomfort or irritation with the serum's use after her laser treatment. She did find that the RGN‐6 serum helped significantly with postprocedure erythema and dryness. Overall, the patient was very satisfied with the treatment regimen and inclusion of the advanced RGN‐6 serum in her postprocedure skincare. Her GAIS score at week 4 was 1, very much improved with the most significant reduction in redness (Figure 5).

### 3.5 Case 5. PicowayLaser Treatment With Advanced RGN‐6 Serum for Dark Spots

**FIGURE 6:**
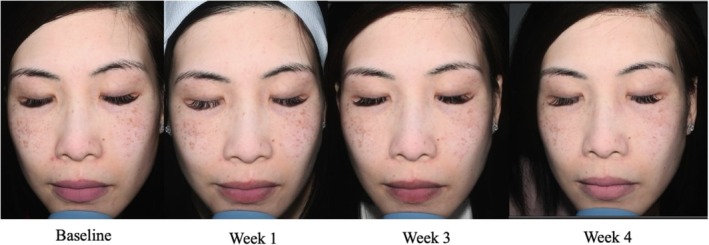
Case 5. A 42‐year‐old woman (Fitzpatrick Skin Type 3). Use of Picoway laser treatment in conjunction with advanced RGN‐6 serum.

A 42‐year‐old woman, FST 3, presented with concerns of skin discoloration and multiple dark spots and sunspots across her face. Physician assigned skin scores at baseline were 0 for fine lines, 4 for skin tone/evenness, 4 for skin clarity, 0 for firmness, 0 for elasticity, 3 for radiance, and 1 for smoothness. At this time, the patient underwent Picoway laser spot treatment over the dyspigmented portions of her face. After the procedure, the patient was instructed to use the advanced RGN‐6 serum twice daily over her whole face. Over the next 4 weeks, the patient did not see any change in her facial dyschromia and saw a 1‐point worsening of her skin tone/evenness (5) and skin clarity (5) at week 4. Her final GAIS score was 4, no change (Figure 6). The approach led to a 1‐point decrease in skin dryness and erythema over the treatment period and had no adverse effects on the patient's skin. While there was minimal improvement in the patient's skin, the patient reported pleasant product texture and a feeling that her skin was smoother and more hydrated with the skincare.

### 3.6 Case 6. Aerolase LightPod Neo With Advanced RGN‐6 Serum for Hyperpigmentation

**FIGURE 7:**
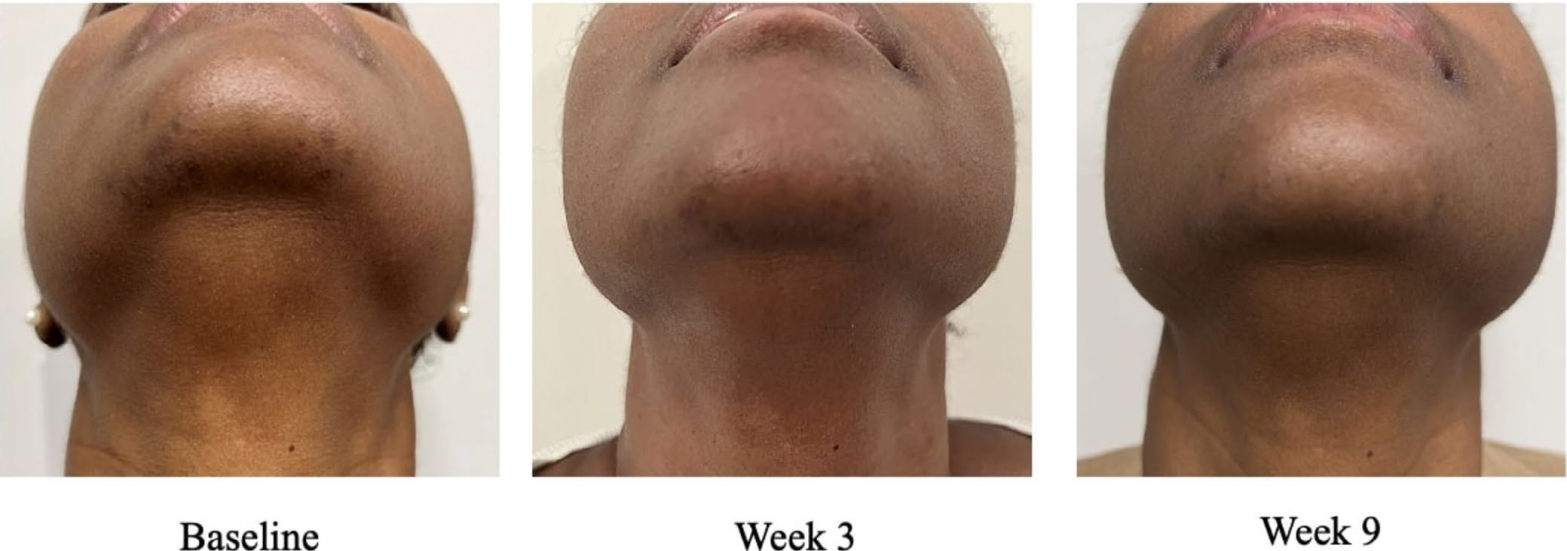
Case 6. A 49‐year‐old woman (Fitzpatrick Skin Type 6). Use of Aerolase LightPod Neo in conjunction with advanced RGN‐6 serum.

A 49‐year‐old woman, FST 6, presented with concerns of hyperpigmentation on her chin. The PIH was a result of chin hair removal, which induced inflammation and PIH over time. At baseline, her physician assigned skin scores were the following: 2 for fine lines, 3 for skin tone/evenness, 3 for skin clarity, 1 for skin firmness, 2 for elasticity, 4 for radiance, and 3 for smoothness. At this time, the patient underwent treatment of her hyperpigmentation with the Aerolase LightPod Neo. The patient was then instructed to use the RGN‐6 serum twice daily on her chin and the rest of her face to restore skin tone evenness. By week 3, the patient had significant improvement in skin radiance and fine lines, skin tone/evenness, skin clarity, firmness, elasticity, radiance, and smoothness with scores of 1, 2, 2, 1, 1, 1, and 2, respectively. Improvement in the hyperpigmentation was also observed (Figure 7). Overall, the patient had a GAIS score of 2 by week 3. The patient continued seeing improvement by week 9 (Figure 6). The patient strongly agreed that the serum provided a healthy glow to her skin and made her skin more even. She did not report any adverse effects of the RGN‐6 serum.

## 4 Discussion

In this case series, the advanced RGN‐6 serum was effectively used in integrated skincare regimens in women with skin of color, ranging from FST 3 to 6, across a wide variety of ages. The advanced RGN‐6 serum contains a patent‐pending formula that effectively targets 6 aspects of skin aging and the skin regeneration process. The novel formulation contains six active ingredients that include 1% eperuline, 0.2% ectoin, 10% glycorepair, 0.2% bioceramide 603, 3% acetyl tetrapeptide‐9, and 2% niacinamide. These ingredients address antioxidant and anti‐inflammation via 1% eperuline and 0.2% ectoin, skin barrier and dermal recovery via 10% glycorepair, 0.2% bioceramide 603, and 3% acetyl tetrapeptide‐9, and PIH/PIE prevention via 2% niacinamide. Eperuline and ectoin are antioxidant and anti‐inflammatory ingredients that have been uncommonly used and combined in skincare. Studies have also shown that ectoin protects cell membranes from dryness by forming a water shell around proteins, thereby leading to increased hydration of the cell membrane and reduced water loss [10]. Thus, the properties of ectoin lead to barrier repair of damaged skin such as that after procedures [10]. Glycorepair is a carob seed extract that has been shown to be involved in epidermal and dermal regeneration and shown to protect elastin from degradation [11]. These effects lead to enhanced facial rejuvenation. Bioceramide 603 is a biomimetic ceramide developed by L'Oreal to reduce melanogenesis and improve barrier repair, thereby targeting dyspigmentation and dryness in aging skin. Acetyl tetrapeptide‐9 improves skin thickness and firmness through stimulation of collagen synthesis [7]. Niacinamide reduces hyperpigmentation, wrinkling, and inflammatory changes in skin [8, 12]. Together, these ingredients target common signs of aging in a multipronged approach.

Experts agreed that the serum was easy to apply and use. Interestingly, throughout the case study, expert dermatologists saw most notable and significant results in redness reduction of patient skin. Experts hypothesized that the novel combination of anti‐inflammatory, ectoin and eperuline ingredients, may be conferring synergistic antiredness effects on postprocedure skin. In lighter skin tones, the effect of the RGN‐6 serum on redness was more easily visualized than on darker skin tones. Patients with darker skin tones more often reported improvement in itching, dryness, and itching rather than redness, which may also be reflective of improving skin inflammation. Further, the serum demonstrated notable effects in targeting wrinkles, fine lines, skin tone/evenness, and radiance. Niacinamide, combined with the other active ingredients (e.g., Bioceramide 603), was also hypothesized to be an important factor in improving PIH in case 6. Overall, the serum was well tolerated in all patients without any reported irritation or adverse events. Experts agreed that the serum was safe to be used in all skin types including acne‐prone and sensitive skin. Patient feedback also reflected high satisfaction rates with the product and patients felt that their skin felt smoother, firmer, and more moisturized after 4 weeks of twice daily use.

In the future, split face studies will be important to evaluate the direct effects of the RGN‐6 serum on redness, wrinkles, and other visible signs of aging separate from procedure‐induced effects. In addition, head‐to‐head studies using RGN‐6 serum and other postprocedure creams such as heparin sulfate or topical corticosteroids may also identify RGN‐6 as an alternative to be used in patients' skincare regimens. Integrated skincare regimens allow for personalization and optimization of a patient's cosmetic procedures. The advanced RGN‐6 serum supports energy‐based and other rejuvenation procedures to enhance patient results and satisfaction with postprocedure care. Recommended integrated skincare treatment guidelines for postprocedure care will be imperative to help guide the future of cosmetic medicine.

## 5 Limitations

This real‐world case series demonstrates the use of the advanced RGN‐6 serum under real‐world conditions in treatment regimens devised by expert dermatologists. The serum is combined with a variety of cosmetic procedures in this case series, making it difficult to distinguish the effects of the procedure from that of the RGN‐6 serum. In addition, these cases do not provide data from a controlled, clinical trial environment. Instead, these cases seek to share expert opinion, clinical experiences, and insight on how to use the serum in clinical practice and in combination with various cosmetic procedures.

## 6 Conclusion

The presented real‐world cases demonstrate the potential benefits of advanced RGN‐6 serum in conjunction with a broad range of cosmetic procedures in a diverse patient population with skin of color. The RGN‐6 serum simultaneously targets the 6 important factors for skin rejuvenation: (1) barrier re‐epithelization, (2) redness and inflammation, (3) cellular energy stimulation, (4) elastin and collagen stimulation, (5) antioxidant, and (6) postinflammatory hyperpigmentation (PIH) through its novel formulation of ingredients. These features complement the targeted effects of various cosmetic procedures that aim at facial rejuvenation. In this case series, the serum was combined with salicylic acid peels, RF microneedling, Picoway laser treatment, Sofwave ultrasound, and Aerolase laser in integrated skincare regimens. The selected cases represented women ranging from ages 28 to 77 years with FST 3 through 6. Overall, expert consensus revealed that the serum was effective in reducing facial erythema and improving skin tone over a 4‐week period. The advanced RGN‐6 did not generate adverse reactions or irritation in any of the represented patient cases. Expert dermatologists' experience with the advanced RGN‐6 serum provides evidence for integrative skin regimens to be safely and effectively used in conjunction with facial rejuvenation procedures. Future studies will focus on quantifying the benefits of optimized skincare for postprocedure care and maximizing patient results.

## Author Contributions

All authors (A.A., R.A.B., P.B., S.G., O.A.I., M.R., H.W.‐L., and V.C.) contributed to developing and conducting real‐world cases, reviewing, and selecting the cases presented in this publication. All authors (A.A., R.A.B., P.B., S.G., O.A.I., M.R., H.W.‐L., and V.C.) contributed to developing the manuscript, reviewing this work, and agreeing with the content.

## Consent

All authors obtained written informed consent from the individuals who participated in the real‐world case series. The participants in the real‐world series allowed the recording of their photographs to be used for the manuscript and its publication.

## Conflicts of Interest

The authors declare no conflicts of interest.

## Data Availability

The data that support the findings of this study are available on request from the corresponding author. The data are not publicly available due to privacy or ethical restrictions.
